# Design expert based optimization of the pyrolysis process for the production of cattle dung bio-oil and properties characterization

**DOI:** 10.1038/s41598-024-57843-z

**Published:** 2024-04-24

**Authors:** Lovepreet Kaur, Jayant Singh, Alaknanda Ashok, Vijay Kumar

**Affiliations:** 1 Department of Farm Machinery & Power Engineering, G B Pant University of Agriculture & Technology, Pantnagar, Uttarakhand 263145 India; 2 Department of Electrical Engineering, G B Pant University of Agriculture & Technology, Pantnagar, Uttarakhand 263145 India; 3 Department of Biosciences, Swami Rama Himalayan University, Dehradun, Uttarakhand 248016 India

**Keywords:** Cattle dung, Bio-oil production, Fixed-bed pyrolysis, Design expert, Energy science and technology, Engineering

## Abstract

This study aimed to optimize pyrolysis conditions to maximize bio-oil yield from cattle dung, a waste product of livestock practices. Pyrolysis of cattle dung was carried out in batch type reactor. The pyrolysis process was optimized using a central composite design in response surface methodology, with conversion parameters such as pyrolysis temperature, vapor cooling temperature, residence time, and gas flow rate taken into account. The cattle dung bio-oil was analyzed using gas chromatography/mass spectroscopy (GC/MS), an elemental analyzer, a pH probe, and a bomb calorimeter. Furthermore, the ASTM standard procedures were used to determine the bio-fuel characteristics. The optimized conditions were found to be a pyrolysis temperature of 402 °C, a vapor cooling temperature of 2.25 °C, a residence time of 30.72 min, and a gas flow rate of 1.81 l min^−1^, resulting in a maximum bio-oil yield of 18.9%. According to the findings, the yield of bio-oil was predominantly affected by pyrolysis temperature and vapor cooling temperature. Moreover, the bio-oil that was retrieved was discovered to be similar to conventional liquid fuels in numerous ways.

## Introduction

In the last several decades, the rapid depletion of conventional fossil fuels, as well as the daily growth in environmental pollution as a result of their extensive use, has prompted a search for alternative renewable and sustainable fuel sources. In this context, biomass derived fuels seem to be the promising path. Biomass includes a wide range of materials in its definition, including forest and agricultural waste, livestock manure, energy crops, as well as organic wastes like sewage sludge, food, and sorted organic waste^1,2^. Livestock manure is particularly intriguing in this regard. They can be considered a resource for renewable energy that is underutilized.

The global production of cattle manure peaked at over 23 million tons per day in 2007^3,4^.In India, approximately 535 million livestock animal units (cattle, buffalo, goats sheep, pigs, poultry and others) are reported in 2019^5^. It has been estimated that India produces approximately 1098 million tons of cattle dung annually. Liquid fossil fuel reserves, on the other hand, are limited. Animal manure contains a high carbon and has adequate physicochemical characteristics; thus, it is a tremendous source of energy. The demand for limited fuels would be greatly reduced if it could be fiscally transformed into bio-oil. Economic conversion of cattle dung to bio-oil can replace a fair amount of fossil fuels.

Cattle dung is used in agricultural fields (with or without composting) for fertilizing the soil to increase the crop productivity^6^. Spreading cattle dung as manure in agricultural fields utilizes its nutritional value; however, organic carbon present in it is ignored. Anaerobic digestion is another way of utilizing the cattle dung. It is a biological process, which converts organic carbon of cattle dung into biogas using anaerobic microorganisms^7^. However, the implementation of anaerobic digesters for the production of biogas is limited by long retention period, necessity of large reactor volumes and the requirement of on-site application of the biogas. Using pyrolysis technologies is an alternative solution for cattle manure as the process involves shorter reaction times^8,9^. Bio-oil produced during pyrolysis process is easy to store and transport. Moreover, pyrolysis is advantageous when raw material (cattle dung) is near to where the application of final product (bio-oil) is required. As cattle dung is a lignocellulosic biomass, organic carbon present in it is utilized for bio-oil production during pyrolysis process with biochar as the by-product. Bio-oil is a mixture of complex organic compounds such as acids, alcohol, ethers, ketones, aldehydes, phenols, esters, sugar, furans, and nitrogen mixes. Apart from energy and fuel, these compounds have the potential to be transformed into higher-value-added products namely resins, liquid smoke, anhydro-sugars (levoglucosan), binders for palletizing and wood preservative^10^. Bio-char, on the other hand, is a solid by-product of the pyrolysis process that can be used in agricultural fields to increase the soil's water and nutrient retention capacity. Bio-char also absorbs carbon from the atmosphere and works as a carbon sink on agricultural land^11^.

Previously, researches have been undertaken to determine the potential of livestock manure as a bio-fuel source. Yin et al.^12^ were possibly the first to carry out hydrous pyrolysis (Hydrothermal liquefaction) of cattle manure for bio-oil production. They studied the effect of conversion temperature, initial conversion pressure, residence time, process gas, and the mass ratio of cattle manure to water on bio-oil yield. According to their research, the amount of bio-oil that can be obtained from cattle manure using hydrothermal liquefaction (HTL) can be affected by the temperature and type of gases used in the process. However, they found that higher initial conversion pressure, longer residence time, and a larger amount of cow manure in relation to water can decrease the yield of bio-oil, since it is converted into gases and char/tar under these conditions. They also discovered that the main non-polar components found in bio-oil were toluene, ethyl benzene, and xylene, which are similar to the components found in crude oil, gasoline, and diesel. Effect of temperature and catalysts on hydrothermal liquefaction of livestock manure has been investigated in numerous studies^13–16^. In their study, Theegala and Midgett^13^ investigated the hydrothermal liquefaction of dairy manure. Their research involved examining two parameters: temperature, with an operational range of 250–350 °C, and the quantity of catalyst (Na_2_CO_3_) tested at levels of 0, 1, 2, 3, and 4 g with each operating temperature. The results showed that the highest bio-oil yield was achieved at 350 °C after 15 min of retention time and with a catalyst quantity of 1 g. Posmanik et al.^14^ conducted a study on the impact of acid and alkali addition on hydrothermal liquefaction of two waste biomass feedstocks – manure digestate and carbohydrate-rich food waste. The HTL reactions were performed at 300 °C for 60 min, both with and without the addition of acid or base. The results indicated a higher impact of acid addition on HTL reactions for manure digestate compared to food waste. The addition of acid resulted in decreased recovery of C1-4 carboxylic acids and increased production of cyclic furan compounds in the aqueous phase. Gas chromatography-mass spectrometry (GC/MS) analysis of bio-crude oil showed that the addition of acid favored dehydration reactions in the HTL media. In their study, Chen et al.^15^ conducted hydrothermal liquefaction (HTL) of dairy manure at 350 °C and investigated the effects of various chemicals, including NH_3_.H_2_O, H_3_PO_4_, and glycerol, on the liquefaction process. They suggested that the addition of these chemicals could serve as a sustainable alternative for dairy manure management. Specifically, the use of NH_3_.H_2_O and H_3_PO_4_ during HTL resulted in a significant increase in the production of liquid chemicals. Moreover, the addition of NH_3_.H_2_O or glycerol led to higher amounts of non-polar toluene, xylene, and other benzene-containing compounds, while H_3_PO_4_ produced high levels of acids, pyridine, 3-methyl-pyridine, 2,6-dimethyl-pyrazine, 2-cyclopenten-1-ones, and phenols. Posmanik et al.^14^ conducted a study on the impact of temperature on the hydrothermal liquefaction (HTL) process of cattle manure at three different temperatures: 200, 250, and 300 °C. The results indicated that the temperature significantly influenced the yield of biocrude oil and hydro char from manure. Hydro char production was favored at the lowest temperature of 200 °C, with a yield of 45.4 ± 0.6%. In contrast, biocrude oil production increased with temperature, yielding 13.5 ± 0.3% and 24.6 ± 2.0% at 250 and 300 °C, respectively. However, the overall conversion yields (i.e., biocrude oil + hydro char) remained similar for all three temperatures, ranging from 47.4 to 55.5%. Jeong et al.^17^ carried out pyrolysis of swine manure. They investigated the yield and properties of biocrude-oil at different pyrolysis temperatures. Highest yield of bio-oil was found to be 18.48 wt% at optimum conditions. Pyrolysis of goat manure was performed by Erdogdu et al.^18^ to study the influence of the temperature on solid, liquid and gas products. The study's findings revealed that goat manure can be used as a valuable raw material for the production of bio-oil.

The idea of this research is to utilize abundantly available cattle dung for bio-oil production in order to meet the higher energy demands. Although, thermogravimetric analysis of cattle dung has been performed, no research on optimizing pyrolysis parameters for cattle dung bio-oil production has been done. The RSM based on the CCD was used to design experiments as well as construct quadratic equation models that predicted the optimum conditions for desired response. In this work, factors such as pyrolysis temperature, vapor cooling temperature, residence time and gas flow rate were optimized to maximize the conversion of condensable gas into bio-oil. The use of a factorial design to determine the effect of these parameters requires a large number of experimental runs, which is both time consuming and expensive. As a result, central composite design (CCD) in RSM was used to carry out only representative experiments to limit the number of experimental runs. In addition, ASTM standard methods and GC/MS analysis were used to determine the fuel and chemical properties of the bio-oil produced to determine its suitability as fuel.

## Material and methods

### Raw material

Cattle dung for the study was collected from Dairy Farm of G B Pant University of Agriculture and Technology, Pantnagar, Uttarakhand, India. Wet samples were then dried in direct sunlight, up to an average moisture content of 8.13%. Using a hammer mill grinder, samples of sun-dried cattle dung were ground into small particles. The grinder was operating on a single-phase electric motor of one horsepower and set to a speed of 1250–1440 rpm. Following that, the ground material was sieved with an IS sieve No. 20 with a perforation size of 0.841 mm. Ground cattle dung had an average particle size of 0.841 mm. The samples were then placed in a container to be used throughout the research period.

### Proximate, elemental and composition analysis

Proximate analysis was conducted to evaluate the moisture, volatile matter, fixed carbon, and ash content of cattle dung. The moisture content, volatile matter, and ash content were assessed using ASTM D 3173, ASTM D 3175, and ASTM D 3174 standards, respectively. By calculating the difference in weight, the fixed carbon content was determined. A CHN analyzer (Vario EL-III Element Analyzer) was used to investigate the C, H, and N. As, the biomass is made up of C, H, N, O, and ash, the difference in weight was used to determine the amount of oxygen in the biomass. Direct methods were used to determine the content of hemicelluloses, lignin, and cellulose in cattle dung^19^. Before analyzing cellulose, hemicelluloses, and lignin in cattle dung, extractives were determined using a Soxhlet extractor.

### High heating value (HHV) and pH

An automated microprocessor-controlled isothermal bomb calorimeter (WISWO Instruments, New Delhi, India) was used to determine the HHV (ASTM D 240) of cattle dung and its bio-oil. In the case of bio-oil, 1.0 g of oil was poured in a pre-weighed crucible, after that crucible was kept in an adiabatic bomb. The sample was then combusted in a calorimetric bomb under oxygen pressure of 3.4 MPa. For cattle dung, a screw press mechanical pelletizer was used to make 1.0 g pellets of finely ground sample. Following the procedure outlined above, this pellet was used to determine the HHV. Each test was performed in triplicates, with the mean value reported. A digital pH metre (EUTECH Instruments pH 700) was used to determine the pH of cattle dung bio-oil.

### GC/MS analysis

Gas chromatography-mass spectrometry (GC/MS) analysis was carried out to identify compounds with low detection limits and to analyze chemical compounds quantitatively. The cattle dung bio-oil was analysed using a Shimadzu QP-2000 Plus GC/MS analyzer with a Rxi—5 ms column (30.0 m × 0.25 mm × 0.25 µm). For preparation of sample, 50 mg of cattle dung bio-oil was dissolved in 10 ml dichloromethane, then filtered through a 0.22-micron filter. The column was kept at 50 °C for 2 min. The temperature was then raised to 210 °C at a rate of 3 °C min^−1^, and then to 280 °C at a rate of 8 °C min^–1^ for 16 min. Helium was used as the carrier gas, having flow rate of 1.21 ml min^–1^.

### Pyrolysis experimental procedure

**Figure 1:**
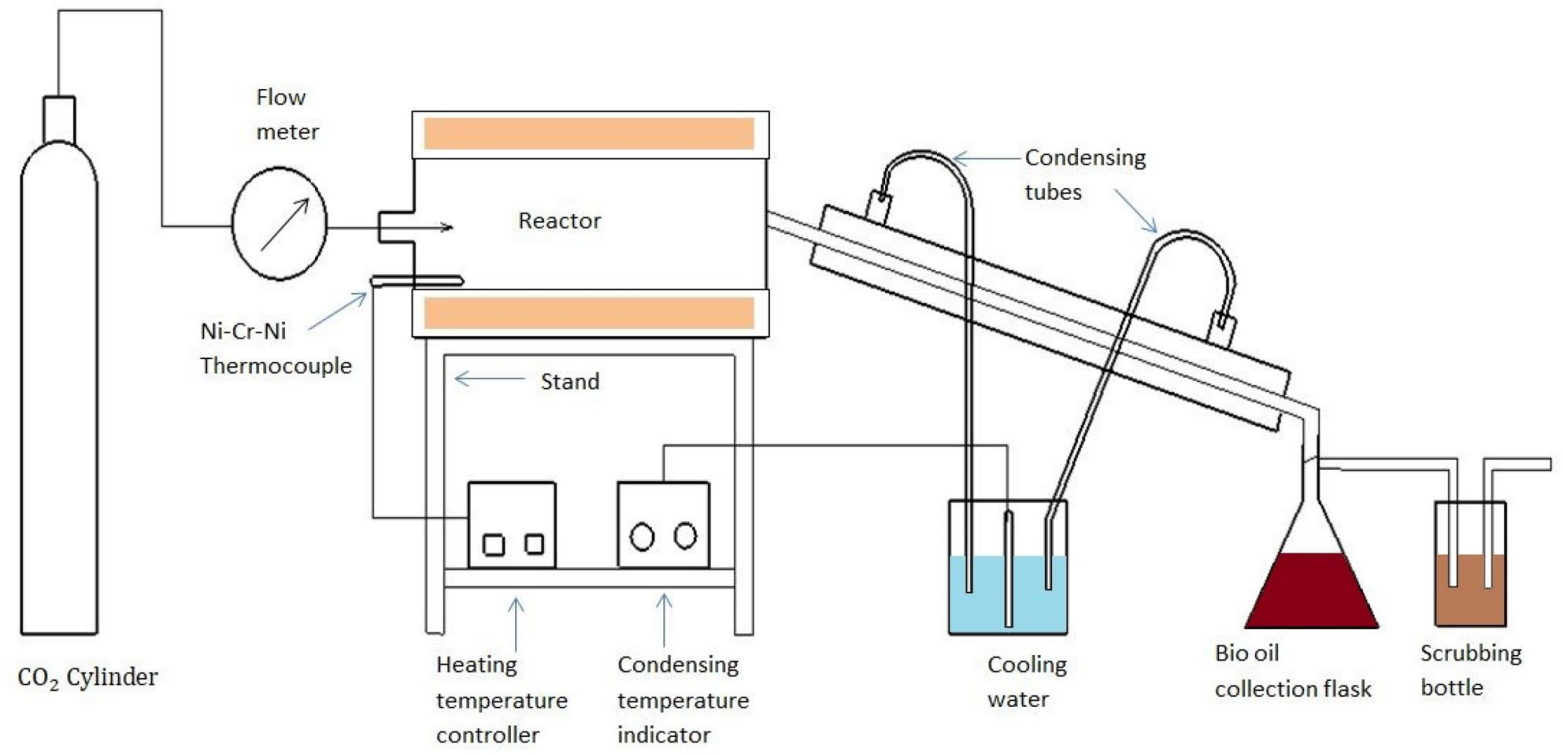
Schematic diagram of laboratory scale bio-oil production unit.

Cattle dung pyrolysis was performed in a batch type reactor. The experimental unit consisted of a stainless-steel tube reactor having internal diameter of 100 mm and length of 400 mm, an electric heater, a carbon dioxide cylinder and vapour condensation unit (Fig. 1). A Ni–Cr–Ni thermocouple was installed inside the reactor to measure temperature. Pyrolysis experiments were carried out in accordance with the central composite design. A total of 198 g of dried cattle dung was fed into the reactor during each run. In the process of loading biomass into the reactor, carbon dioxide (CO_2_) is purged inside the reactor. CO_2_ was continuously introduced through injection point into the reactor to maintain a positive pressure and ensure that reactive or undesirable gases are pushed out. The flow rate for this purging is set to 3 l min^−1^. After the initial purging, the CO_2_ flow rate is adjusted to the required flow rate based on the experimental design. The flow rate of CO_2_ is measured using a gas flow meter with a range of 0.1–3 l min^−1^ and a least count of 0.1 l min^−1^. This adjustment is crucial for controlling the conditions within the reactor. Once the CO_2_ flow rate has been set to the required level, it is allowed to stabilize. Stabilization ensures a consistent and controlled environment inside the reactor. The required temperature was set with the help of temperature controller, after the gas flow rate has reached a steady state. The vapor was quickly cooled by a tube–tube heat exchanger that circulated ice-cold water at the desired temperature. The condensate which is consisted of two phases: aqueous and organic phase, was collected in a beaker. The bio-oil from this mixture was extracted in dichloromethane^11^.

### Experimental design

This study uses experimental design and process optimization tools to maximize the cattle dung bio-oil yield. Process optimization was carried out by employing central composite design (CCD) in response surface methodology (RSM) using Design Expert software (Version 11.1.0.1). The independent variables were pyrolysis temperature (A), vapor cooling temperature (B), residence time (C) and gas flow rate (D). The only response studied was bio-oil yield. Functional relationship between these numerical factors was also determined. Experiments for pyrolysis of cattle dung were carried out in accordance with the central composite design of response surface. There was a total of 30 experimental runs for four variables utilizing CCD, with 16 factorial points, 8 axial points, and 6 central point replications. The mathematical model was developed for optimization and prediction of cattle dung bio-oil yield. Numerical optimization was performed to find out conditions of pyrolysis parameters for maximum bio-oil yield.

## Results and discussion

### Cattle dung composition and characteristics

Properties of cattle dung, obtained by proximate, elemental and compositional analysis are shown in Table 1. As the initial moisture content of collected cattle dung was too high for grinding, the moisture content of wet cattle dung was reduced to less than 10% by drying. It had higher volatile matter content, fixed carbon and lower ash content. Condensable vapor and permanent gases released during biomass heating are termed as volatile matter.

**Table 1:** Physicochemical properties of sun-dried cattle dung.

Proximate analysis (wt %)	
Moisture content	8.13
Volatile matter	72
Ash content	7.7
Fixed carbon	12.13
Ultimate analysis (wt %)	
Cellulose	22.4
Hemi cellulose	17.8
Lignin	12.7
Extractives	47.0
Elemental analysis (wt %)	
C	45.87
H	5.9
N	1.75
S	Not traceable
O	46.47
H/C	1.522
O/C	0.762
Empirical formula	CH_1.522_N_0.032_O_0.762_
HHV (MJ kg^−1^)	17.6

Chouhan and Sarma^20^ explained that higher volatile matter content results in increased amount of bio-oil production during pyrolysis. Chutia et al.^21^ opined that biomass which contains low ash and high volatile content is a suitable material for thermo-chemical conversion. The ultimate analysis results show that cattle dung have an oxygen/carbon (O/C) mole ratio of 0.762 and a hydrogen/carbon (H/C) molar ratio of 1.52. These values are in line with another biomass.

Extractive content in cattle dung was found to be 47.0%. During pyrolysis, Wang et al.^22^ discovered that the extractives can boost bio-oil production while also preventing the formation of char and gas. Bio-oils derived from the biomass with less extractives have more oxygen and less alkane concentration than from its parent material. In another investigation, higher extractive content proven to be reduced production of CO_2_, CO and aldehydes as well as the activation energy while increasing acid production^23^.

Cellulose, hemicelluloses and lignin content for cattle dung were found as 22.4, 17.8 and 12.7%, respectively. Thus, it can be noted that the higher amount of cellulose and hemicelluloses was present in cattle dung compared to lignin. During pyrolysis process, hemicelluloses and cellulose present in biomass results in formation of bio-crude, however lignin produces the higher percentage of solid char ^24,25^. Fahmi et al.^26^ suggested that the average molecular weight and viscosity of bio-oils may increase with higher lignin content, while the water content of the bio-oils decreases. Pyrolysis of cellulose and hemicelluloses at higher temperatures yields less bio-oil because these fibers decompose at lower temperature range. This implies that for increased bio-oil production, cattle dung may require a medium temperature. Higher heating value of cattle dung was determined to be 17.6 MJ kg^−1^. In comparison to other biomass, reported by Mohan et al.^27^ and Torri et al.^28^, HHV of cattle dung was higher.

### Codified quadratic model equation for bio-oil yield

Table 2 presents bio-oil yield from main pyrolysis experimental runs based on Design Expert input. It is clear from the table that bio-oil yield varied from 7.7 to 18.9 wt.% of cattle dung. Highest yield was found in experimental runs 28, 13 and 19. Jeong et al.^17^ observed bio-oil yield of 18.48% from swine manure which had a volatile content of 53.62%. Kim et al.^29^ found that the bio-oil production from poultry litter was 15–30 wt% at temperature range of 450–550 $\mathrm{^\circ{\rm C} }$ which is low in comparison to the bio-oils derived from wood (34–42 wt%). Comparing these values this can be concluded that the yield of bio-oil from batch pyrolysis of cattle dung was satisfactory.

**Table 2:** Design matrix using CCD and bio-oil yield.

Experimental run	A: Pyrolysis temp (°C)	B: Vapour cooling temp (°C)	C: Residence time (min)	D: Gas flow rate (l min^−1^)	Bio-oil yield (%)
1	300	2	15	3	9.2
2	400	6	25	1	16
3	300	6	25	2	13
4	500	10	15	1	14.5
5	400	6	25	2	17.2
6	500	2	35	3	15.5
7	500	6	25	2	15.7
8	300	10	35	3	8.1
9	300	2	15	1	13.5
10	500	2	35	1	17.2
11	500	2	15	3	14.8
12	300	10	15	1	7.8
13	400	6	25	2	17.9
14	500	10	35	3	10.9
15	400	6	15	2	16.7
16	300	10	15	3	7.7
17	400	10	25	2	13.6
18	300	2	35	3	14.5
19	400	2	25	2	17.8
20	300	2	35	1	15.5
21	300	10	35	1	8.9
22	400	6	25	3	13.8
23	400	6	25	2	17.1
24	400	6	25	2	17.6
25	500	2	15	1	16.7
26	400	6	25	2	16.7
27	500	10	15	3	12.8
28	400	6	35	2	18.9
29	500	10	35	1	13.8
30	400	6	25	2	17.5

To justify the validity of the models, analysis of variance (ANOVA) was used. Table 3 summarizes the ANOVA statistics for the quadratic bio-oil yield model. The R^2^ value for bio-oil yield model was found 0.9733. Consequently, the independent variables analyzed accounted for 0.9733% of the overall variance in the findings. The p-value and F-value for each variable had been used to determine its significance at a given degree of confidence. The minimum level of significance that may be utilized to reject null hypothesis, H_0_, is the p-value. As a result, the smaller the value, the greater the significance of the associated parameter and its impact to the dependent variable^30^. According to the ANOVA in Table 3, several of the factors were significant to the regression model, as evidenced by the high F-value. Table 3 shows that four linear factor terms (A, B, C, D), three quadratic terms (A^2^, B^2^, D^2^), and three interaction factors (AC, AC, BC) had the highest effect on cattle dung bio-oil yield at a 95% confidence level, as indicated by the low p-value (0.05) and high F-value.

**Table 3:** ANOVA for response surface quadratic model of coded values.

Source	Sum of squares	Df	Mean square	F-value	p-value	Comment
Model	305.54	14	21.82	39.04	< 0.0001	Significant
A	63.09	1	63.09	112.86	< 0.0001	Significant
B	74.42	1	74.42	133.12	< 0.0001	Significant
C	5.12	1	5.12	9.16	0.0085	Significant
D	15.31	1	15.31	27.38	0.0001	Significant
AB	4.00	1	4.00	7.15	0.0173	
AC	6.50	1	6.50	11.63	0.0039	Significant
AD	0.2500	1	0.2500	0.4472	0.5138	
BC	5.76	1	5.76	10.30	0.0058	Significant
BD	0.7225	1	0.7225	1.29	0.2734	
CD	0.1600	1	0.1600	0.2862	0.6005	
A^2^	15.78	1	15.78	28.22	< 0.0001	Significant
B^2^	3.24	1	3.24	5.79	0.0295	
C^2^	2.50	1	2.50	4.47	0.0516	
D^2^	9.53	1	9.53	17.04	0.0009	Significant
Residual	8.39	15	0.5591			
Lack of fit	7.49	10	0.7492	4.19	0.0636	Not significant
Pure error	0.8933	5	0.1787			
Cor total	313.93	29				
Std. Dev	0.7477		R^2^	0.9733		
Mean	14.36		Adjusted R^2^	0.9484		

**Figure 2:**
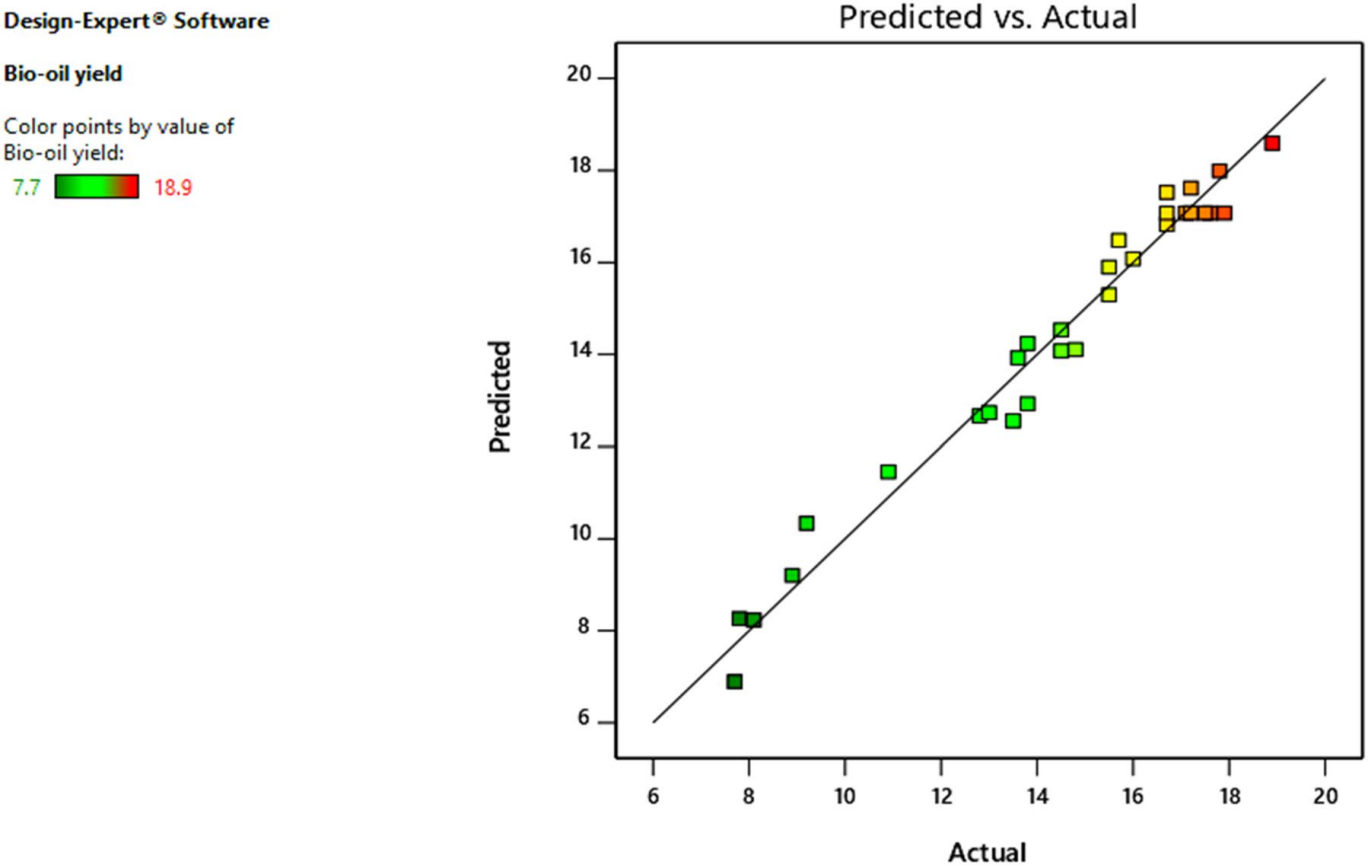
Model predicted value against actual value of bio-oil yield.

**Figure 3:**
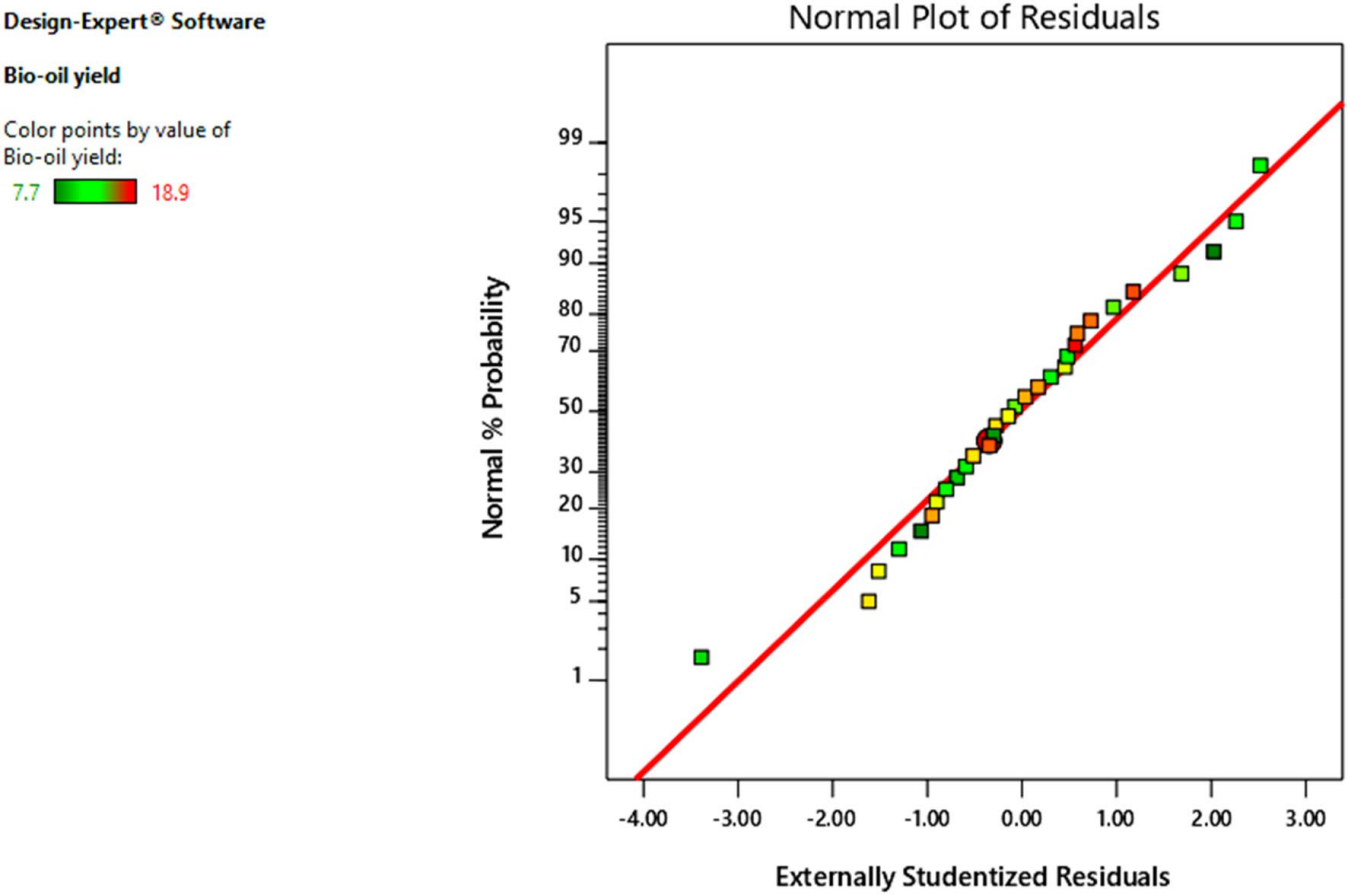
Normal plot distributions of the residuals for bio-oil yield.

Each of the remaining terms had a p-value greater than 0.05, indicating that their impact on the response model was not statistically significant at the 95% confidence level. To put it another way, only model terms with p-values less than 0.05 were found to be significant in the model equation. Statistical analysis (p-values) indicates that bio-oil yield is primarily influenced by pyrolysis temperature (A) and vapor cooling temperature (B). Furthermore, the gas flow rate (D) was found to have a moderate effect on the bio-oil yield, whereas residence time (C) had the least effect. These findings suggest that controlling the range of pyrolysis temperature and vapor cooling temperature is crucial for determining bio-oil yield. Conversely, altering the range of gas flow rate and residence time has a relatively minor impact on bio-oil yield. Therefore, it can be inferred that the pyrolysis temperature and vapor cooling temperature, should be given priority in the optimization of bio-oil yield. The comparison of observed response values acquired from experimental work and projected response values based on the quadratic model is shown in Fig. 2, demonstrating that the model adequately covers the experimental range of research. The normal probability distributions for the studentized residuals for bio-oil yield are shown in Fig. 3. The residuals had a normal distribution, and the points were distributed in a straight line.

However, even with normal data, a certain spread was to be expected; therefore, it could be assumed that the data in the responses of certain models are normally distributed. The model was determined to be fit because the lack of fit test was insignificant. The final empirical model in terms of coded factors (parameters) is given in Eq. (1) based on the ANOVA data and statistical parameters:

1 $$ \begin{aligned} {\text{Bio - oil yield }}\left( {{\text{wt}}\% } \right) & = 17.08 + 1.87A - 2.03B + 0.5333C - 0.9222D + 0.5000AB - 0.6375AC \\ & \quad - 0.1250AD - 0.6000BC + 0.2125BD + 0.1000CD - 2.47A^{2} - 1.12B^{2} + 0.9825C^{2} \\ & \quad - 1.92D^{2} . \\ \end{aligned} $$

where, A, B, C and D are the coded values of pyrolysis temperature, vapour cooling temperature, residence time and gas flow rate, respectively.

The process optimization for the experiments was carried out in order to determine the optimal conditions for maximum yield of bio-oil. Each variable's minimum and maximum ranges, as well as the model's projected responses based on surface and contour plots, were presented. Table 4 shows the constraints for each variable. The Design Expert software suggests different solutions for optimum solutions for maximum yield of bio-oil, which are displayed in Table 5. It illustrates that for the same bio-oil yield, there are three solutions with a desirability of 1. The solution number 1 (pyrolysis temperature of 402 $\mathrm{^\circ{\rm C} }$, vapour cooling temperature of 2.25 $\mathrm{^\circ{\rm C} }$ , residence time of 30.72 min and gas flow rate of 1.81 l min^−1^) was considered optimum. The average bio-oil yield was determined to be 18.9% in confirmation tests using these optimum values. This indicates that the yield obtained under optimal conditions was in good accordance with the model's projected value. Bio-oil yield (18.9%) obtained at optimum conditions is more than twofold of minimum yield (7.7%) obtained without optimization (Table 2).

**Table 4:** Constraints of each variable for optimization of bio-oil yield.

Name of variable	Goal	Lower limit	Upper limit
Pyrolysis temperature	Is in range	− 2	2
Vapour cooling temperature	Is in range	− 2	2
Residence time	Is in range	− 2	2
Gas flow rate	Is in range	− 2	2
Bio-oil yield	Maximize	7.7	18.9

**Table 5:** Optimal conditions for maximal bio-oil yield.

No.	Pyrolysis temperature (°C)	Vapour cooling temperature (°C)	Residence time (min)	Gas flow rate (l min^−1^)	Bio-oil yield (%)	Desirability
1	402.0	2.25	30.72	1.81	18.9	1
2	402.0	2.25	30.72	1.82	18.9	1
3	403.5	2.25	30.72	1.81	18.9	1

### Conversion parameters

**Figure 4:**
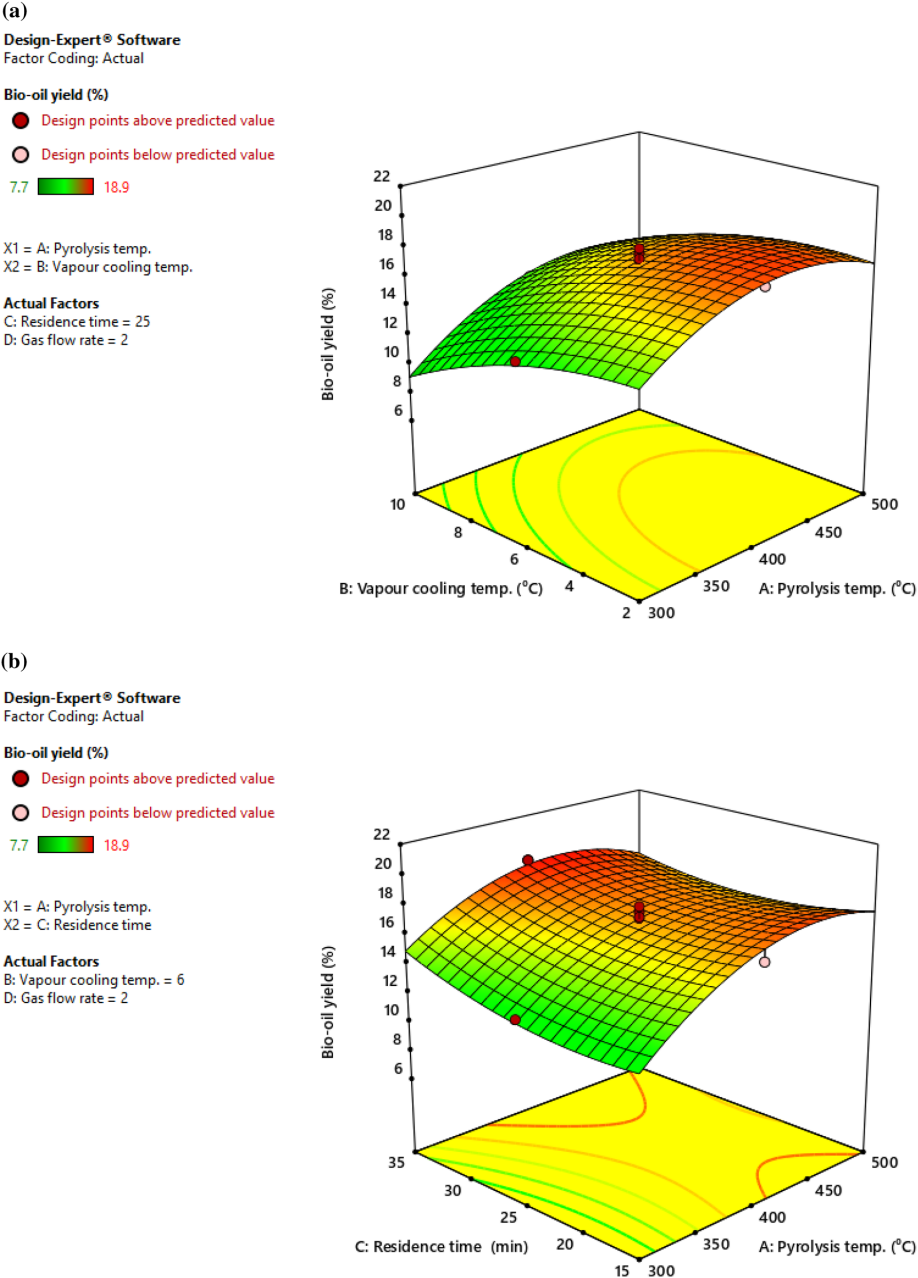

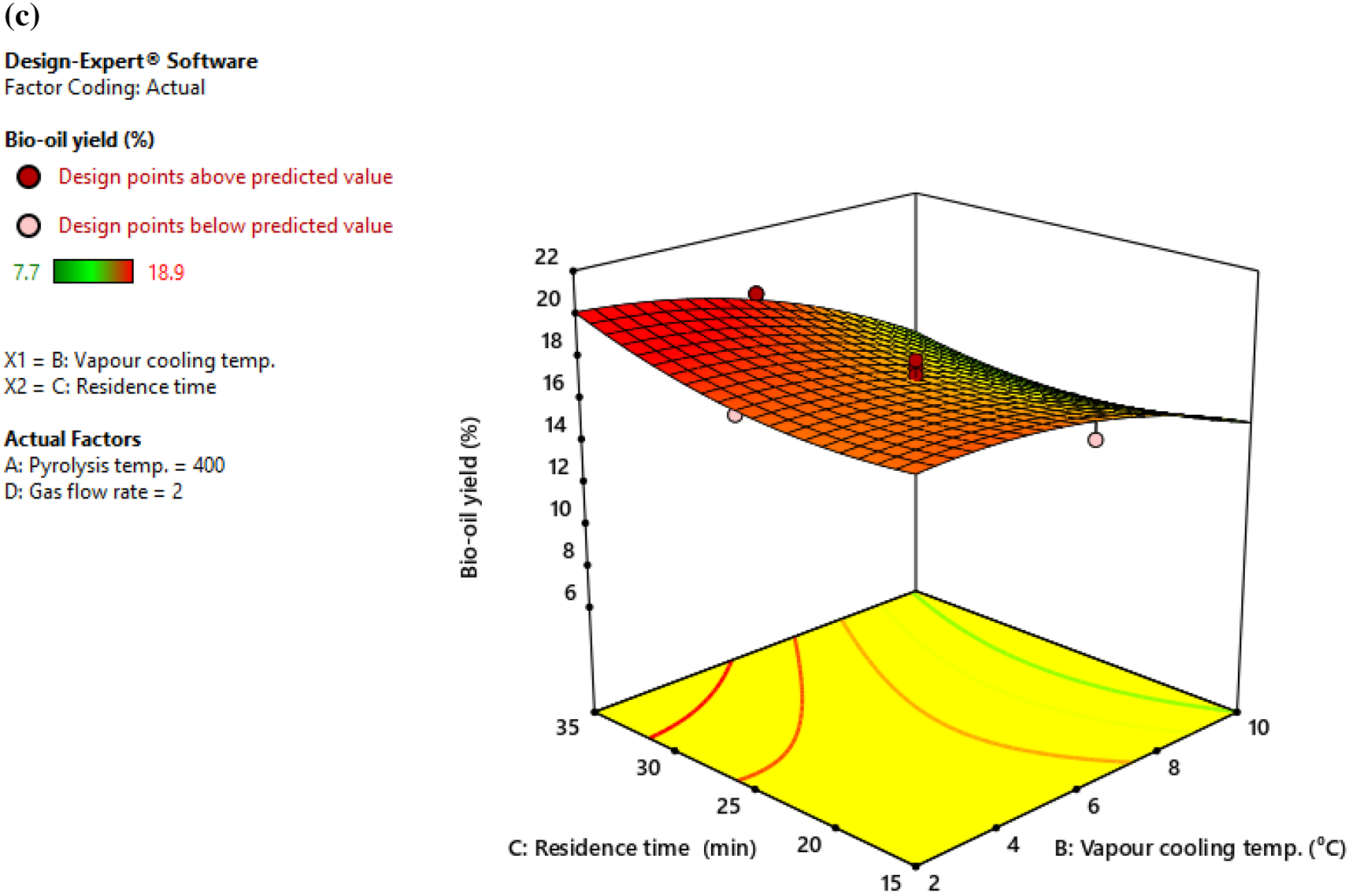
(**a**). Interaction effect of pyrolysis temperature (A) and vapour cooling temperature (B) on bio-oil yield. (**b**). Interaction effect of pyrolysis temperature (A) and residence time (C) on bio-oil yield. (**c**) Interaction effect of vapour cooling temperature (B) and residence time (C) on bio-oil yield.

Effects of four pyrolysis parameters on bio-oil yield from cattle dung have been depicted in Fig. 4. Pyrolysis temperature and vapor cooling temperature were shown to be crucial in obtaining higher oil yield from cattle dung. It indicates that as the temperature is raised, the production of bio-oil increased initially, but then decreased after 400 °C. Bio-oil yield increased with decreasing vapor cooling temperature. It is evident from observations that when residence time reduced, bio-oil yield decreased as well. Thus, above a 25 min residence duration, the maximum yield of bio-oil was found. Therefore, it is evident that higher yield of bio-oil from cattle dung can be attained with a temperature near 400 $\mathrm{^\circ{\rm C} }$ at low vapour cooling temperature and longer biomass residence time. In the present investigation, pyrolysis temperature and vapor cooling temperature had the greatest impact on bio-oil yield. Kelkar et al.^31^ used spent coffee grounds and reported similar results. Similar findings were found by Mohan et al.^27^ in their detailed review work on bio-oil and its characteristics. Beis et al.^32^ suggested that, to prevent subsequent reactions, the vapor should be cooled to ambient temperature right away. The results of this investigation show that a very low vapor cooling temperature is not required for increased liquid recovery.

### Characterization of bio-oil

**Figure 5:**
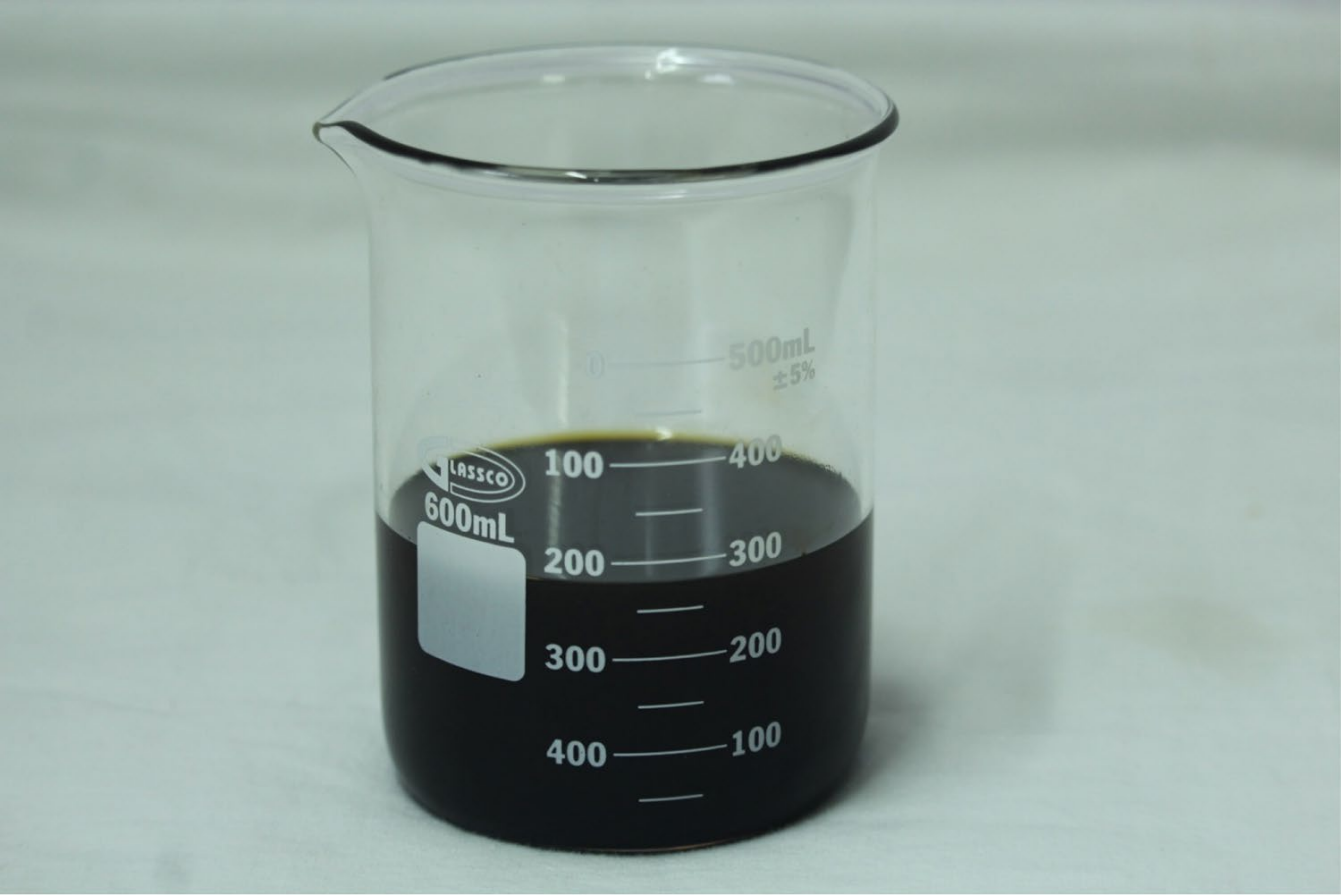
Cattle dung bio-oil.

Figure 5 shows a direct photo of the bio-oil derived from cattle dung under optimal condition of pyrolysis parameters. The cattle dung bio-oil was a dark brown liquid. Fuel properties of cattle dung bio-oil are presented in Table 6.

**Table 6:** Properties of cattle dung bio-oil.

Properties	Standard test methods	Bio-oil	HSD
Appearance		Dark brownish	**–**
Density, 15 °C (g cm^−3^)	ASTM D1217-15	1.028	0.831
pH	pH meter	4.5	5.8
Cloud point (°C)	ASTM D2500	**–**	1
Pour point (°C)	ASTM D5853-05	− 15	-6
Flash point (°C)	ASTM D93	50	54
Fire point (°C)	ASTM D93	58	60
Kinematic viscosity (cSt)	Redwood viscometer	3.2	2.93
Ash content, %	ASTM D 482	0.775	–
C residue, %	ASTM D189	10.73	0.004
HHV, MJ kg^−1^	ASTM D 240	30.68	45.34
Elemental analysis (wt %)			
C		58.91	90.0
H		7.42	9.50
N		2.54	0.06
O		31.13	0.3
H/C		1.49	–
O/C		0.397	–
Empirical formula		CH_1.50_N_0.036_O_0.396_	–

The pH of cattle dung bio-oil was found 4.5. When considering bio-oil as a fuel, the pH is one of the most essential characteristics because it signals corrosiveness. The bio-oils are acidic in nature due to the presence of organic acids, like acetic acid, carboxylic acid and formic acid. When the pH of bio-oil drops, the oil becomes more acidic. It has been found that in most bio-oils, the pH value is in the range of 2–4. However, the pH of cattle dung bio-oil was higher which was in the same line, results reported by Park et. al. ^33^ and Greenhalf et. al. ^34^ with rice straw and wheat straw respectively. This might be owing to the fact that cow dung bio-oil contains less acidic compounds. The pH of high-speed diesel (HSD) ranges between 5.5 and 8 which is slightly higher than the pH of cattle dung bio-oil. The density of bio-oil was observed 1.028 g cm^−3^ indicating that it is heavier than that of high-speed diesel (HSD), of which density is 0.840 g cm^−3^. Another key fuel property is kinematic viscosity that determines its atomization characteristic. The value of kinematic viscosity of bio-oil at 40 $\mathrm{^\circ{\rm C} }$ was 3.2cSt. These values were close to HSD (2–5 cSt) suggesting that it may be utilized, without any changes to the injection mechanism in compression ignition engines.

Its pour point, flash point and fire point were found to be − 15 $\mathrm{^\circ{\rm C} }$, 50 $\mathrm{^\circ{\rm C} }$ and 58 $\mathrm{^\circ{\rm C} }$, respectively. Flash point and fire point were near to conventional HSD having these values of 54 $\mathrm{^\circ{\rm C} }$ and 60 $\mathrm{^\circ{\rm C} }$, respectively. However, pour point was much lower than HSD. Due to its lower pour point, it can be used in winter-grade diesel that can withstand extremely low temperatures during the winter months enabling the fuel to remain liquid. The cloud point could not be assessed due to the dark color. High heating value of cattle dung bio-oil was found to be 30.68 MJ kg^−1^. Value of HHV was almost 70% of HSD and much higher than its parent material, cattle dung. Similar findings were reported by Saikia et al.^35^ and Mandal et al.^36^ in bio-oil prepared from Arundo donax and pine needles, respectively.

Carbon, hydrogen, nitrogen, and oxygen content of cattle dung bio-oil were found to be 58.91, 7.42, 2.54, and 31.13% respectively. In comparison to feedstock, oxygen content in bio-oil was less. Bordoloi et al.^11^ observed that low oxygen content of bio-oil was beneficial as high oxygen content was not advantageous to transportation fuel production. Further, higher amount of hydrogen content indicates presence of higher number of hydrocarbons in bio-oil, which indicates that it is suitable for transportation fuel generation. Empirical formula of bio-oil was typical of biomass pyrolysis oil and close to HSD of which empirical formula is C_12_H_24_.

The chemical compounds in cattle dung bio-oil were identified by GC/MS analyses. Mohan et al.^27^ reported that bio-oils are complex blends of diverse organic components from different chemical groups. Chemical properties of bio-oil are dependent on the pyrolysis process parameters and the characteristics of the source material. Total 43 chemical compounds were identified in bio-oil. The concentrations of these compounds are shown in Table 7. The compounds identified were from seven major organic groups and mainly composed of ketones (35.08%) followed by alcohol (14.43%), anhydride (13.12%), phenol (11.38%), esters (8.44%), pyridine and its derivatives (4.76%), and furan (4.37%). Wang et al.^37,38^ reported the same results in their studies. Ketones and anhydride present in bio-oil can be upgraded to fuel through co-cracking^39^ and transestrification^40^, respectively. However, esters and alcohol could be directly utilized as transport fuel. Extraction of phenols from the bio-oil may be economically viable from the point of view of industrial use. Pyridine and its derivatives are valuable and rare compounds which are used for DNA characterization. The extraction of these compounds may yield greater bio-oil value.

**Table 7:** Quantitative analysis of main compounds in cattle dung bio-oil by GC/MS.

S. N.	Retention time (min)	Compound	Chemical formula	Chemical group	Concentration (%)
1	4.29	2-Picoline	C_6_H_7_N	Pyridine derivatives	2.62
2	4.72	2-Cyclopentenone-1	C_5_H_6_O	Ketone	6.76
3	4.89	2-Methylcyclopentan-1-one	C_6_H_10_O	Ketone	0.56
4	5.04	Di(2-ethylbutyl) ether	C_12_H_26_O	Ether	0.21
5	5.14	Hexanoic acid	C_6_H_12_O_2_	Acid	0.50
6	5.50	Allyl-butanoate	C_7_H_12_O_2_	Ester	0.21
7	5.74	2-Furylmethanol	C_5_H_6_O_2_	Alcohol	7.88
8	5.95	1,2-Ethanediol diacetate	C_6_H_10_O_4_	Ester	5.95
9	6.17	2,4-Hexadienal	C_6_H_8_O	Aldehyde	0.19
10	6.42	2,6-Lutidine	C_7_H_9_N	Pyridine derivatives	0.39
11	7.22	2-Methyl-2-cyclopenten-1-one	C_6_H_8_O	Ketone	5.18
12	7.46	2-Acetyl-furan	C_6_H_6_O_2_	Furan	1.28
13	7.60	3-Oxo-1-butenyl 2-methylpropanoate	C_8_H_12_O_3_	Ester	0.77
14	7.95	Adipic ketone	C_5_H_8_O	Ketone	4.83
15	8.34	2-Cyclohexen-1-one	C_6_H_8_O	Ketone	0.33
16	8.45	2,5-Dimethylpyridine	C_7_H_9_N	Pyridine	0.34
17	8.95	2,3-Dimethylpyridine	C_7_H_9_N	Pyridine	0.82
18	9.48	4-Hydroxypentanoic acid lacton	C_5_H_8_O_2_	Furanone	0.38
19	9.93	3-Methyl-2-cyclopentenon	C_6_H_8_O	Ketone	4.25
20	10.07	2-Oxobutyl acetate	C_6_H_10_O_3_	Ester	0.62
21	11.15	4,4-Dimethyl-2-cyclopenten-1-one	C_7_H_10_O	Ketone	0.61
22	11.30	2,3-Dimethylcyclopent-2-en-1-one	C_7_H_10_O	Ketone	0.45
23	11.65	Phenyl alcohol	C_6_H_6_O	Alcohol	6.55
24	11.84	n-Butyric anhydride	C_8_H_14_O	Anhydride	13.12
25	13.47	Corylon	C_6_H_8_O_2_	Ketone	9.98
26	14.02	(6*E*)-3,5-Dimethyl-1,6-octadiene	C_10_H_18_	Alkene	0.27
27	14.35	1-Oxacyclohexan-2-one	C_5_H_8_O_2_	Ketone	0.31
28	15.14	ortho-Cresol	C_7_H_8_O	Phenol	1.54
29	15.92	4-Methoxyphenol	C_7_H_8_O_2_	Phenol	5.24
30	17.12	2-Amino-5,6-dihydro-4,4,6-trimethyl-4H-1,3-oxazine	C_7_H_14_N_2_O	Oxazine	0.97
31	17.69	2-Hydroxy-3-ethyl-2-cyclopenten-1-one	C_7_H_10_O_2_	Ketone	1.54
32	19.28	11-Fluoroundecyl trimethylsilyl ether	C_14_H_31_FOSi	Ether	0.38
33	20.74	para-Ethylphenol	C_8_H_10_O	Phenol	1.44
34	23.69	2,3-Dihydro-benzofuran	C_8_H_8_O	Benzofuran	2.71
35	24.89	4-Ethyl guaiacol	C_9_H_12_O_2_	Phenol	0.48
36	25.38	Hex-1-en-3-yl acetate	C_8_H_14_O_2_	Ester	0.34
37	26.71	4-Vinyl-guaiacol	C_9_H_10_O_2_	Phenol	0.42
38	28.35	2,6-Dimethoxyphenol	C_8_H_10_O_3_	Phenol	2.06
39	29.76	5,5-Diethyl-4-methylene-1,2-dioxolan-3-one	C_8_H_12_O_3_	Ketone	0.29
40	43.45	Desaspidinol	C_11_H_14_O_4_	Phenol	0.20
41	45.59	5,10-Diethoxy-2,3,7,8-tetrahydro-1H,6H-dipyrrolo[1,2-a:1ʹ,2ʹ-d]pyrazine	C_14_H_22_N_2_O_2_	Pyrazine	0.21
42	49.42	Phthalic acid, bis(2-ethylhexyl) ester (6CI,8CI)	C_24_H_38_O_4_	Ester	0.69

## Conclusions

In this study, CCD/RSM of Design Expert® was adopted to determine the optimum conditions of conversion parameters to obtain maximum bio-oil yield from cattle dung using a batch type reactor. The optimum conditions for pyrolysis of cattle dung at 1.00 desirability were pyrolysis temperature of 402 °C, a vapour cooling temperature of 2.25 °C, a residence time of 30.72 min, and a gas flow rate of 1.81 l min^−1^. The quadratic model fit the experimental data adequately, and the analysis of variance exhibited a high coefficient of determination for bio-oil yield (R^2^ = 0.973). Under optimum conditions, the maximum bio-oil yield was observed to be 18.9% and had a heating value of 30.68 MJ kg^−1^. Bio-oil yield from pyrolysis of cattle dung was significantly dependent on pyrolysis temperature and vapor cooling temperature. GC/MS analysis of bio-oil detected presence of ketones, alcohol, anhydride, phenol, esters, pyridine and its derivatives, and furan. In many aspects, the fuel and chemical properties of bio-oil from cattle dung were comparable to conventional transportation fuels. Results of research suggest that the production of bio-oil from cattle dung can be scaled up, in bio-refinery concept.

## Data Availability

All data generated or analyzed during this study are included in this published article.
